# Deep learning enables automatic detection of joint damage progression in rheumatoid arthritis—model development and external validation

**DOI:** 10.1093/rheumatology/keae215

**Published:** 2024-04-10

**Authors:** Mikko S Venäläinen, Alexander Biehl, Milja Holstila, Laura Kuusalo, Laura L Elo

**Affiliations:** Turku Bioscience Centre, University of Turku and Åbo Akademi University, Turku, Finland; Department of Medical Physics, Turku University Hospital, Turku, Finland; Turku Bioscience Centre, University of Turku and Åbo Akademi University, Turku, Finland; Department of Radiology, University of Turku and Turku University Hospital, Turku, Finland; Centre for Rheumatology and Clinical Immunology, Division of Medicine, University of Turku and Turku University Hospital, Turku, Finland; Turku Bioscience Centre, University of Turku and Åbo Akademi University, Turku, Finland; Institute of Biomedicine, University of Turku, Turku, Finland

**Keywords:** rheumatoid arthritis, radiographic joint damage, radiographic scoring, disease progression, deep learning

## Abstract

**Objectives:**

Although deep learning has demonstrated substantial potential in automatic quantification of joint damage in RA, evidence for detecting longitudinal changes at an individual patient level is lacking. Here, we introduce and externally validate our automated RA scoring algorithm (AuRA), and demonstrate its utility for monitoring radiographic progression in a real-world setting.

**Methods:**

The algorithm, originally developed during the Rheumatoid Arthritis 2–Dialogue for Reverse Engineering Assessment and Methods (RA2-DREAM) challenge, was trained to predict expert-curated Sharp–van der Heijde total scores in hand and foot radiographs from two previous clinical studies (*n* = 367). We externally validated AuRA against data (*n* = 205) from Turku University Hospital and compared the performance against two top-performing RA2-DREAM solutions. Finally, for 54 patients, we extracted additional radiograph sets from another control visit to the clinic (average time interval of 4.6 years).

**Results:**

In the external validation cohort, with a root mean square error (RMSE) of 23.6, AuRA outperformed both top-performing RA2-DREAM algorithms (RMSEs 35.0 and 35.6). The improved performance was explained mostly by lower errors at higher expert-assessed scores. The longitudinal changes predicted by our algorithm were significantly correlated with changes in expert-assessed scores (Pearson’s *R *=* *0.74, *P* < 0.001).

**Conclusion:**

AuRA had the best external validation performance and demonstrated potential for detecting longitudinal changes in joint damage. Available from https://hub.docker.com/r/elolab/aura, our algorithm can easily be applied for automatic detection of radiographic progression in the future, reducing the need for laborious manual scoring.

Rheumatology key messages

- A novel algorithm for automated scoring of RA-related joint damage, AuRA, was introduced.
- AuRA demonstrated good external validity and capability for detecting longitudinal changes in joint damage.
- Ultimately, AuRA can enable automatic disease monitoring and facilitate the use of laborious radiographic scores.

## Introduction

RA is a chronic inflammatory disease with a global prevalence of 0.25% [1]. RA is characterized by a symmetrical synovial joint inflammation causing pain, swelling and stiffness in the affected joints. If left untreated, the inflammation can lead to permanent substantial damage to bone and articular cartilage leading to functional impairment and loss of mobility as the disease progresses. As widely acknowledged, although the course of RA is unique to each individual patient, early diagnosis and treatment initiation improve long-term outcomes significantly and prevent otherwise irreversible disability [2, 3].

Conventional radiography is recommended as the principal imaging technique for detecting initial joint damage and its accrual in RA [4]. The Sharp–van der Heijde score (SvH), which evaluates joint-space narrowing and bone erosions, is currently the standard scoring method to assess and follow structural damage in radiographs of hands, wrists and feet in clinical trials [5]. Regardless, the careful assessment of multiple individual joints using the SvH method is very labour-intensive and requires expertise, limiting the use of SvH for research purposes. Automated tools to assist with SvH scoring would improve its usability not only in research but eventually in routine clinical practice as well.

Deep learning algorithms have demonstrated substantial potential in a variety of biomedical problems, including radiographic scoring in RA [6, 7]. Recently, the Rheumatoid Arthritis 2–Dialogue for Reverse Engineering Assessment and Methods (RA2-DREAM) challenge was organized as a worldwide crowdsourcing effort to develop algorithms with the highest achievable performance for automated assessment of SvH scores from radiographs of hands and feet [8]. Under evaluations against bootstrapped data and an independent post-challenge dataset, the winning algorithms managed to produce scores close to expert-curated scores, demonstrating that automatic scoring of RA radiographs is feasible and has potential for further development into clinical applications.

Despite the recognized potential of deep learning in estimating radiographic scores, the sensitivity of the available algorithms to detect and monitor longitudinal changes in joint damage, one of the key aspects of radiographic scoring in RA, is still largely underreported. Regardless, it has been suggested that deep learning could be able to detect even subtle changes of disease and therefore allow novel possibilities for early therapy and prevention of the development of clinically significant disease [6]. Overall, the ability to accurately detect radiographic changes would be beneficial not only in clinical practice by providing additional evidence of progression for the treating rheumatologist when discussing treatment options with the patient, but also in clinical trials monitoring the efficacy of DMARDs via changes in radiographic joint damage scores, most often SvH [6, 9].

In the present work we describe our automatic RA scoring algorithm (AuRA) developed during the RA2-DREAM challenge, as well as its post-challenge improvements. We evaluated the performance of our algorithm in an independent validation cohort from a local university hospital and compared it with top-performing RA2-DREAM solutions. Finally, we applied AuRA to evaluate longitudinal changes in joint damage in a subset of patients from the independent validation cohort to demonstrate the utility of our algorithm in monitoring joint damage over time.

## Methods

### Study sample

**Figure 1.**
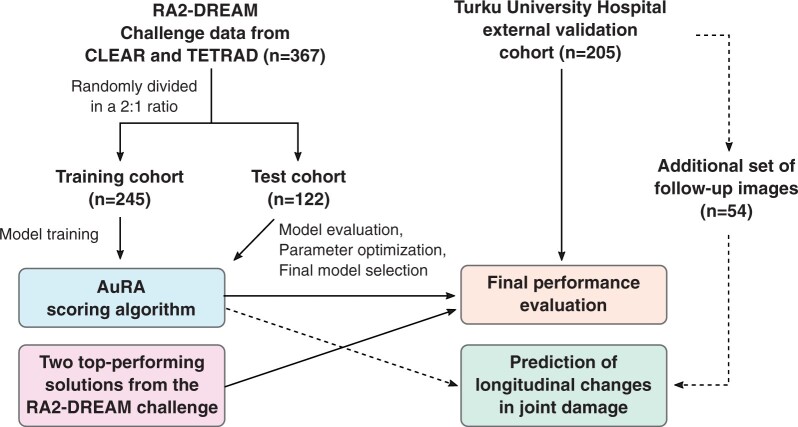
Overview of the patient cohorts included and study workflow. RA2-DREAM: Rheumatoid Arthritis 2–Dialogue for Reverse Engineering Assessment and Methods; CLEAR: Consortium for the Longitudinal Evaluations of African Americans with Early Rheumatoid Arthritis; TETRAD: Treatment Efficacy and Toxicity in Rheumatoid Arthritis Database and Repository; AuRA: automated RA scoring algorithm

Overview of the study workflow and the use of different datasets is provided in Fig. 1. Model development was carried out using the RA2-DREAM challenge data consisting of high-resolution radiographs from two previous National Institutes of Health–funded studies led by investigators of University of Alabama at Birmingham: CLEAR (Consortium for the Longitudinal Evaluations of African Americans with Early Rheumatoid Arthritis) [10] and TETRAD (Treatment Efficacy and Toxicity in Rheumatoid Arthritis Database and Repository) [11, 12]. For external model validation and evaluation of longitudinal changes in joint damage, we collected an independent dataset consisting of RA patients from Turku University Hospital, Finland.

#### Model development cohort

In total, the data for model development included radiographic image sets from 367 patients with associated expert-curated SvH scores collected in CLEAR and TETRAD. Each image set contained posteroanterior radiographs of left and right hands and feet, totaling four images per image set. The joint-space narrowing scores for individual joints ranged between 0 and 4 for all joints (21 in total, 15 joints located in hands/wrists and 6 joints located in feet), whereas the erosion scores ranged between 0 and 5 for 16 different sites located in each hand/wrist and 0 and 10 for 6 sites in each foot. These individual scores were summed together to yield the total SvH scores ranging from 0 to 448. During our internal model training and testing, we split the image sets into separate training (*n* = 245, 67% of the data) and test cohorts (*n* = 122, 33% of the data).

#### External validation cohort

Using electronic health record data, we identified RA patients aged at least 18 years who visited the rheumatology clinic of Turku University Hospital, Finland, in 2013–19. Among these, we selected a sample of patients (*n* = 205) with varying degrees of joint damage for whom we extracted posteroanterior hand and foot radiographs used for clinical monitoring of the disease. The SvH scoring was performed similarly as in the model development cohort. Additionally, for 54 patients, we extracted a second set of radiographic images from a follow-up visit to the clinic. All extracted radiographs were scored by an experienced musculoskeletal radiologist.

#### Research ethics

In the RA2-DREAM challenge, the use of challenge data from CLEAR and TETRAD was approved by the University of Alabama at Birmingham Institutional Review Board and the requirement for informed consent was waived according to 45 CFR 46.116. The analysis of Turku University Hospital data was approved by the institutional review board of Turku University Hospital (T110/2020). According to Finnish legislation on the secondary use of health data, no ethical committee approval or written consent was required due to the retrospective register-based study design. All patient data were fully pseudonymized prior to accessing them and the analyses were carried out in a secure data analysis environment to ensure full data protection.

### Automated SvH scoring algorithms

Overall, we applied four different solutions for automatic assessment of total SvH scores from extracted radiographic image sets: the algorithm we (team Aboensis V) originally submitted for the RA2-DREAM challenge, an improved version of our algorithm referred to as AuRA and two top-performing RA2-DREAM solutions. All solutions were packed into Docker images and applied to external validation data on a secure data analytics platform with 4 CPU cores running at 2.3 GHz and 16 GB of memory available for analyses.

#### Original RA2-DREAM algorithm by team Aboensis V

Here, we provide only a brief summary of steps taken to make predictions for each image set in our original solution. Full description of the algorithm, including used computational software and applied parameters, can be found in the Supplementary Material, available at *Rheumatology* online as well as in our method writeup available on Synapse.org. In short, the basic workflow of the algorithm consisted of five main steps: (i) preprocessing including adaptive histogram equalization and resizing images to square shape, (ii) joint detection using you only look once (YOLO) v3 object detection algorithm [13], (iii) refined joint labelling using gradient boosting machine multi-class classifiers, (iv) score prediction using the YOLO v3 algorithm and (v) score post-processing including imputation of missing scores for undetected joints using median predicted scores. Our original algorithm is available on Synapse.org with accession number syn21680240.

#### AuRA

**Figure 2.**
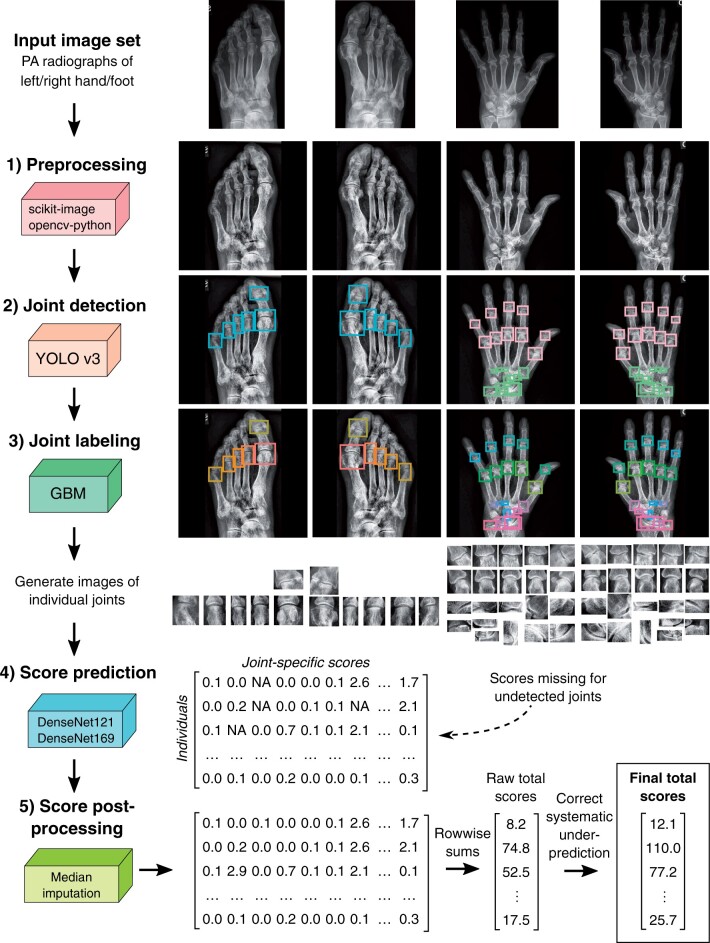
Overview of the automated RA scoring algorithm (AuRA) algorithm for automatic scoring of radiographic joint damage in RA. YOLO: you only look once; GBM: gradient boosting machine

Due to underperformance of our original algorithm observed during the post-submission phase, we carefully revised all underperforming components into the updated version entitled AuRA. Full description of key modifications in relation to the original solution are available in the Supplementary Material, available at *Rheumatology* online. In essence, the basic workflow of AuRA remained the same, but the main difference between the two algorithms was the replacement of YOLO v3 score prediction with convolutional neural network models based on either the DenseNet121 or the DenseNet169 [14] architecture that predicted the joint-specific scores as continuous values instead of discrete classes. During training of these models, we also applied data augmentation techniques to increase the diversity of the training set. Furthermore, during score post-processing, we performed imputation of missing joints with median values among joints with high Pearson correlation or similar anatomical location instead of using all narrowing or erosion scores predicted for an individual (Supplementary Fig. S1A, available at *Rheumatology* online). Finally, due to systematic underprediction of total SvH scores based on simple summation of joint-specific scores, we multiplied the obtained raw total scores with a scaling factor determined using the model development data, to obtain the final total SvH scores (Supplementary Fig. S1B, available at *Rheumatology* online). To improve the clinical utility of AuRA, we also added an additional feature to generate individual patient reports for the predictions. The overall workflow of AuRA is visualized in Fig. 2.

#### Algorithms by top-performing teams in the RA2-DREAM challenge

In addition to our original RA2-DREAM and AuRA solutions for automatic assessment of SvH scores, we applied solutions from two top-performing teams in the RA2-DREAM challenge: Gold Therapy and Team Shirin. In the final scoreboard, the solution by Gold Therapy was ranked first for the prediction of joint-wise erosion scores and second for the prediction of joint-wise narrowing scores, whereas the solution by Team Shirin was ranked first for predicting the overall damage. Detailed description of each of the solutions and Docker images required for running them are available on Synapse.org with accession numbers syn21499370 and syn21478998 for Gold Therapy and Team Shirin, respectively.

### Evaluation of algorithm performance and detection of longitudinal changes

First, we applied all four algorithms to obtain the predicted total SvH scores for the patients in the Turku University Hospital external validation cohort to compare the performances of the solutions. We primarily assessed the performance of each algorithm by comparing the scores reported by each method to the expert-assessed scores in terms of root mean square error (RMSE) defined as


$$\mathrm{RMSE}=\sqrt{\frac{1}{N}\sum_{i=1}^{N}{\left(x_{i}-{\hat{x}}_{i}\right)}^{2}}$$

where $x_{i}$ is the expert-assessed SvH score for subject i, ${\hat{x}}_{i}$ is the predicted SvH score, and N is the total number of subjects. We also used coefficient of determination *R*^2^ as a secondary performance measure


$$R^{2}=1-\frac{\sum_{i=1}^{N}{\left(x_{i}-{\hat{x}}_{i}\right)}^{2}}{\sum_{i=1}^{N}{\left(x_{i}-\bar{x}\right)}^{2}}$$

where $\bar{x}$ is the mean of observed SvH scores. To visualize the relationships between predicted and expert-assessed SvH scores, we drew scatter plots with linear trendlines. To study the agreement between predicted and expert-assessed while accounting for systematic over- or underestimation of effects, we calculated Lin’s concordance correlation coefficient (CCC) for each method. We also used Bland–Altman plots to study systematic errors in the predicted scores.

On the level of individual joints, we evaluated the sensitivity of AuRA in finding the relevant anatomical regions of interest (ROIs) by comparing the predictions against manually selected ground-truth ROIs. To assess the performance of AuRA in estimating scores for individual joints while establishing total SvH scores, we calculated RMSEs and Pearson correlation coefficients for comparisons against ground-truth scores.

Finally, we applied AuRA to predict the total SvH scores for the 54 additional image sets in the external validation cohort to evaluate the potential of quantifying longitudinal changes in joint damage, defined as difference in total SvH scores between two consecutive image sets. We visualized the longitudinal changes in reference expert-assessed SvH scores and time differences between the analyzed visits using a spaghetti plot. The relationship between changes in predicted and expert-assessed SvH scores was assessed using Pearson correlation and visualized using a scatter plot with linear trendline. The systematic errors between predicted and expert-assessed changes were visualized using a Bland–Altman plot.

The statistical analyses were carried out using R statistical computing environment version 4.0.3 (R Core Team, 2016; R: A language and environment for statistical computing, R Foundation for Statistical Computing, Vienna, Austria; https://www.R-project.org/). R package *ggplot2* [15] was used for visualization of results. The level of significance was set at *P* < 0.05.

## Results

### Patient characteristics

Demographic and clinical characteristics of the RA patients included in training and validation cohorts are summarized in Table 1. The patients in the training cohort were on average 55 years old, majority were women (84%) and seropositive (74% positive for RF). Similarly, the patients in the Turku University Hospital external validation cohort were on average 60 years old, mostly women (78%), and predominantly seropositive (89% RF and/or ACPA positive). In both cohorts, the individual joints generally exhibited abundant low bone erosion and joint narrowing scores in both hands (Supplementary Fig. S2A, available at *Rheumatology* online) and feet (Supplementary Fig. S2B, available at *Rheumatology* online), thus resulting in positively skewed total SvH score distributions (Supplementary Fig. S2C, available at *Rheumatology* online). In the training cohort, the total SvH scores ranged between 0 and 390 with a median value of 13 [interquartile range (IQR) 34], whereas in the validation cohort, the scores had slightly better coverage across higher total scores and ranged between 0 and 342 with a median value of 41 (IQR 82). The group of patients selected for longitudinal analysis were representative of the total sample in the external validation cohort (Table 1).

**Table 1.** Demographic and clinical characteristics of the patients in training and validation cohorts

Characteristic	Training data		Validation data			
	RA2-DREAM		Turku University Hospital			
	All patients (*n* = 367)		All patients (*n* = 205)		Longitudinal follow-up (*n* = 54)	
SvH score, median (IQR)						
Total	13	(34)	41	(82)	33	(63)
Joint-space narrowing	7	(21)	32	(45)	28	(32)
Erosion	5	(15)	7	(39)	7	(41)
Age at first radiographic examination (years), mean (s.d.)	54.9	(13.2)	60.0	(13.8)	56.1	(13.3)
Sex, *n* (%)						
Men	60	(16.3)	46	(22.4)	13	(24.1)
Women	307	(83.7)	159	(77.6)	41	(75.9)
Diagnosis^a^, *n* (%)						
Seropositive RA	273	(74.4)	182	(88.8)	47	(87.0)
Seronegative RA	90	(24.5)	23	(11.2)	7	(13.0)
NA	4	(1.1)	0	(0.0)	0	(0.0)
DAS28(-CRP), mean (s.d.)	NA	NA	2.7	(1.2)	2.7	(1.2)
HAQ	NA	NA	0.8	(0.7)	0.7	(0.5)

a For training data, seropositivity shown for rheumatoid factor. In training data, only 250 patients were positive for anti–cyclic citrullinated peptide antibodies. RA2-DREAM: Rheumatoid Arthritis 2–Dialogue for Reverse Engineering Assessment and Methods; SvH: Sharp–van der Heijde; IQR: interquartile range; NA: not available; DAS28: DAS assessing 28 joints.

### Prediction of individual joint scores

Overall, AuRA detected the relevant ROIs for joint-specific scoring with a sensitivity of 93.3% (95% CI 92.8–93.7%) (Supplementary Table S1, available at *Rheumatology* online). In general, the joint detection performed better for joints with lower SvH scores (Supplementary Fig. S3, available at *Rheumatology* online) and for images with high joint detection rates (Supplementary Fig. S4, available at *Rheumatology* online). The scores estimated for individual joints varied according to task and anatomical location (Table 2). With an RMSE of 0.72 (range 0.48–0.92), the best performance was achieved for the estimation of erosion scores in the finger joints. In contrast, in terms of absolute values, the worst performance was observed for the estimation of erosion scores in the foot joints with an RMSE of 1.67 (range 1.28–2.02). However, it should be noted that they also had the widest range of possible values. In the estimation of narrowing scores, the performance was similar in all anatomical locations with pooled RMSEs of 1.06, 0.98 and 1.03 for finger, wrist and foot joints, respectively.

**Table 2.** Performance of AuRA in predicting individual joint scores in terms of RMSE and Pearson’s *R*

Joint group	Erosion				Narrowing			
	(Scale: 0–5 for hands, 0–10 for feet)				(Scale: 0–4)			
	RMSE	(range)	*R*	(95% CI)	RMSE	(range)	*R*	(95% CI)
Finger	0.72	(0.48–0.92)	0.61	(0.60–0.63)	1.06	(0.66–1.32)	0.68	(0.66–0.69)
Wrist	0.87	(0.69–1.04)	0.76	(0.74–0.77)	0.98	(0.91–1.12)	0.68	(0.66–0.70)
Foot	1.67	(1.27–2.02)	0.78	(0.76–0.79)	1.03	(0.85–1.38)	0.65	(0.62–0.67)

AuRA: automated RA scoring algorithm; RMSE: root mean square error.

### Prediction of total SvH scores

**Figure 3.**
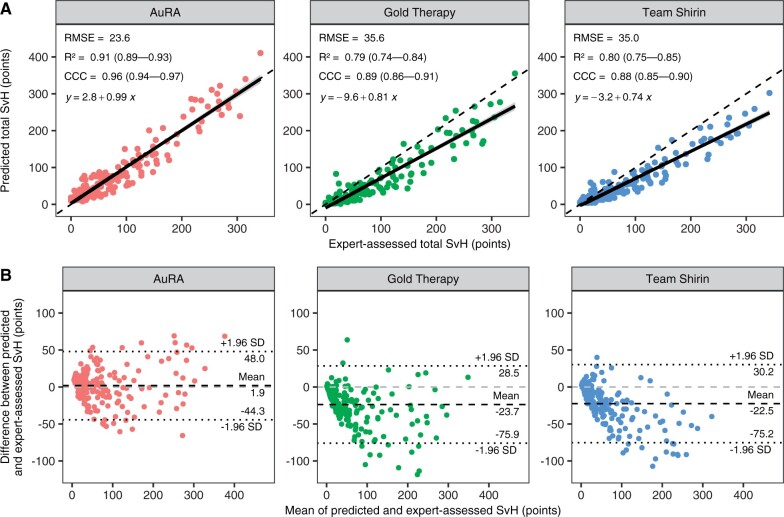
Performances of the evaluated algorithms in the external validation cohort. (**A**) Scatter plots with linear trend lines and (**B**) Bland–Altman plots illustrating the agreement between predicted and expert-assessed Sharp–van der Heijde (SvH) scores. RMSE, CCC and SD denote root mean square error, Lin’s concordance correlation coefficient and standard deviation, respectively. Values between brackets indicate 95% CIs. AuRA: automated RA scoring algorithm

In the validation cohort, AuRA had the best correspondence with the expert-assessed total SvH scores with an RMSE of 23.6, *R*^2^ of 0.91 (95% CI 0.89–0.93) and CCC of 0.96 (95% CI 0.94–0.97) (Fig. 3A). The top-performers from the RA2-DREAM challenge, Gold Therapy and Team Shirin, produced slightly higher RMSEs of 35.6 and 35.0, lower *R*^2^ of 0.79 (95% CI 0.74–0.84) and 0.80 (95% CI 0.75–0.85), as well as lower CCCs of 0.89 (95% CI 0.86–0.91) and 0.88 (95% CI 0.85–0.90), respectively. The difference was explained by systematic underprediction of SvH scores at greater expert-assessed scores, characterized by linear trendline slopes of 0.81 and 0.74, compared with 0.99 with AuRA. As a result, algorithms by Gold Therapy and Team Shirin predicted on average 23.7 (95% CI –28.5 to 75.9) and 22.5 (95% CI –30.2 to 75.2) points lower total SvH scores compared with expert-assessed scores, respectively (Fig. 3B). With AuRA, the deviation was on average only 1.9 (95% CI –44.3 to 48.0) points. Due to predicting the joint-specific scores as discrete classes, our original Aboensis V algorithm performed poorly, resulting in an RMSE of 80.4 and *R*^2^ of –0.06 (Supplementary Fig. S5, available at *Rheumatology* online).

### Longitudinal changes in total SvH scores

**Figure 4.**
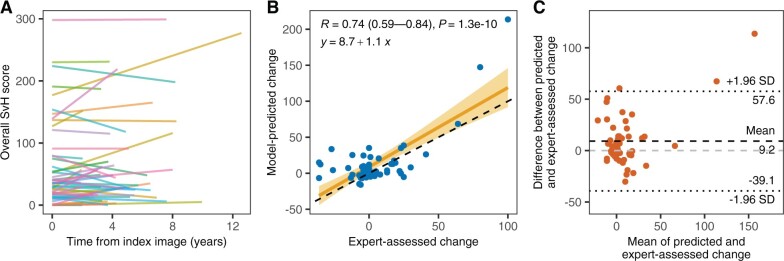
Prediction of longitudinal changes in total Sharp–van der Heijde (SvH) scores using AuRA. (**A**) Longitudinal changes in expert-assessed SvH scores *vs* time from index image for patients in the external validation cohort included in the follow-up analysis (*n* = 54). (**B**) Scatter plot showing the relationship between model-predicted and expert-assessed changes in SvH scores together with Pearson correlation and its 95% CI as well as equation for the line of best fit. (**C**) Bland–Altman plot illustrating the agreement between predicted and expert-assessed changes in SvH scores. AuRA: automated RA scoring algorithm

For patients in the validation cohort with an additional follow-up image set, the average time difference between the two visits was 4.6 years (range 1.9–12.6). During that time period, the total SvH scores increased on average by 5.3 points (range –36 to 100) (Fig. 4A). The longitudinal changes estimated with AuRA correlated significantly with the expert-assessed scores (Pearson’s *R *=* *0.74, 95% CI 0.59–0.84, *P* < 0.001), indicating a strong positive relationship between the two measures (Fig. 4B). The linear trendline fitted to the results revealed some error especially on intercept, leading on average to 9.2 points (95% CI –39.1 to 57.6) greater changes predicted by AuRA for two consecutive image sets compared with the expert-assessed changes (Fig. 4C).

## Discussion

In the present study, we introduced and externally validated AuRA, our automated solution for quantification of RA-related joint damage in hand and foot radiographs. In the external validation cohort, AuRA produced well-calibrated estimations of total SvH scores and outperformed two top-performing solutions from the RA2-DREAM challenge, a worldwide crowdsourcing effort for developing high-performance methods for this task. Furthermore, the longitudinal changes in SvH scores estimated with AuRA correlated significantly with expert-assessed changes in SvH scores, indicating suitability for automatic monitoring of disease progression.

Deep learning–based algorithms enable automatic quantification of radiographic joint damage in terms of clinically relevant but laborious SvH scores starting from raw, unprocessed radiographs. In the best-case scenario, they can provide invaluable support for diagnostics and clinical decision-making, requiring minimal user effort [8]. Here, using a computer configuration comparable to a standard workstation available at any modern clinic, all evaluated algorithms scored the external validation data with an average processing time of <2 min for the four images of a single patient. By enabling graphical processing unit (GPU) computing, computation times could have been reduced further down to only seconds. In addition to low costs, deep learning–based estimations have greater reproducibility compared with manual, observer-dependent scoring. Therefore, if successful, they could easily be applied to improve statistical power of future research studies by providing scores for large amounts of already existing radiographic imaging data in electronic health records or new data collected in clinical trials aiming to identify novel, more efficient DMARDs [6, 8].

An important aspect in improving the performance of AuRA was to apply a scaling factor to the raw total SvH scores calculated from individual joint predictions, effectively reducing systematic underprediction of total SvH scores. The underprediction observed with the competing approaches, as well as with our method when this step was excluded, occurred most likely due to the positively skewed distributions of SvH scores in both training and validation cohorts, thus putting more emphasis on predicting the lower scores more accurately during the training phase. Our validation cohort had also a wider spectrum of SvH scores revealing the underperformance at higher scores more effectively. The positively skewed score distributions, however, are in line with scores reported in the literature [16, 17], suggesting that the consideration of this score imbalance might be an important factor when attempting to predict accurate scores for the higher end of SvH spectrum using deep learning approaches.

Due to the increasing number of effective treatment options for RA, fewer patients have severe disease and accrue radiological damage, making the signal of disease progression harder to detect in the presence of noise arising from intra- and interobserver variability [18, 19]. In our patient material, we demonstrated that the radiographic changes estimated with AuRA had a strong relationship with expert-assessed changes, therefore suggesting potential in automatic identification of patients with progressive disease based on their follow-up images. Overall, the predicted changes had fairly large error margins and included a notable degree of systematic bias. This could, however, be explained by the small number of patients included in the follow-up cohort and the use of only one trained expert scorer. Regardless, the modest overall increase observed for total SvH scores in this cohort fell within the range of previously reported annual progression rates of 0–3 units [17, 20–22] indicating a good representation of different progression profiles.

To estimate the SvH scores, AuRA performs various intermediate steps, most importantly detection and identification of individual joints from input radiographs. These steps are performed with excellent accuracy in AuRA and as such, could also be used for computer-aided diagnosis by automatically detecting and highlighting the corresponding anatomical ROIs for subsequent manual scoring, or other processing, of the joints in both hands and feet. Previous approaches performing this task have typically focused only on hand radiographs but the reported sensitivities of ∼95% are comparable to our present performance [23–25].

Despite the potential of deep learning–based automated scoring highlighted in this work, it should be noted that this represents the initial evaluation of AuRA against external validation data, and the algorithm is still at a very preliminary stage, especially in terms of clinical applications. In particular, the prediction errors were noticeably large also for individuals with lower SvH scores which would be the case for most patients in routine clinical practice as depicted by the positively skewed distributions. This may be partially attributed to the relatively small sample sizes in both training and validation cohorts which were limited by the substantial amount of manual work required even by trained professionals to score radiographs using the SvH method to establish reference scores. Therefore, it would be beneficial to externally validate AuRA and present findings against additional patient cohorts and scores established by multiple expert scorers, using both baseline and follow-up images. Furthermore, by training the models with a larger number of patients and with even a broader spectrum of SvH scores, it is anticipated that the performance would further improve. The optimization of individual models in the algorithm can easily be continued when more data become available and does not require complete restart of model training.

In conclusion, AuRA, our algorithm for automatic quantification of radiographic joint damage in RA according to the SvH method, demonstrated good external validity and potential for monitoring disease progression based on posteroanterior radiographs of hands and feet. The algorithm is freely available as a Docker image and can readily be applied to score any existing datasets. In the future, AuRA could provide timely, accurate data on radiological progression in RA for use in daily clinical practice and has the potential to improve outcomes for patients with RA.

## Supplementary Material

keae215_Supplementary_Data

## Data Availability

Our automated RA scoring algorithm, AuRA, is available as a Docker container here: https://hub.docker.com/r/elolab/aura. However, the original data used for developing and validating the algorithm cannot be made openly available. According to the terms and conditions of the RA2-DREAM challenge, the challenge data cannot be made available in any form and any queries regarding the challenge data should be addressed to the challenge organizers. The external validation data derived from patient health records of Turku University Hospital are also confidential and, in adherence to the EU General Data Protection Regulation (GDPR) and Finnish legislation concerning sensitive data, the authors are not authorized to share the data. However, access to the external validation data may be applied from Auria Clinical Informatics with research permission granted by the Wellbeing services county of Southwest Finland.
