# Supplementary material for: Deep learning enables automatic detection of joint damage progression in rheumatoid arthritis—model development and external validation
Source: Rheumatology (Oxford). 2024 Apr 10;64(3):1068–76. doi: 10.1093/rheumatology/keae215 (PMC11879318; doi:10.1093/rheumatology/keae215)
Supplement: keae215_Supplementary_Data [file keae215_supplementary_data.docx]

# Supplementary Material

**Deep Learning Enables Automatic Detection of Joint Damage Progression in Rheumatoid Arthritis - Model Development and External Validation**

Mikko S Venäläinen, Alexander Biehl, Milja Holstila, Laura Kuusalo, Laura L Elo

This document contains supplementary information and data regarding study cohorts and our prediction algorithms developed for automatic prediction of total Sharp van der Heijde (SvH) scores from posteroanterior radiographs of left and right hands and feet.

# Methods

## The original algorithm and AuRA

Here, more detailed descriptions of our algorithms for automated estimation of total SvH scores from radiographic images of hands and feet are provided. The original algorithm was developed by our team, AboensisV, in the Rheumatoid Arthritis 2–Dialogue for Reverse Engineering Assessment and Methods (RA2-DREAM) challenge whereas the revised algorithm, entitled AuRA, was an improved variation of the submitted algorithm developed during the post-submission phase. Both solutions essentially consisted of the five main steps: 1) preprocessing, 2) joint detection, 3) joint labeling, 4) score prediction and 5) score postprocessing. The main differences between the two solutions are described in the following sections briefly explaining each step. Both approaches were primarily implemented in Python version 3.7.10 except for joint detection, which was implemented using Darknet Open Source Neural Networks in C and CUDA, and joint labeling, which was implemented using R statistical computing environment version 3.6.3. Full description of our original approach is also available on Synapse.org with accession number syn21680240.

### ***Preprocessing***

All input images were initially resized to 800×800 pixels by rescaling the maximum number of pixels along the longer axis to 800 and, if needed, padding the remaining dimension equally on both sides of the image with zeros. Finally, the contrast of the resulting image was enhanced with adaptive histogram equalization. Python packages scikit-image (0.19.3) and opencv-python (4.1.2) were used for preprocessing.

### ***Joint detection***

In order to extract joints for score prediction, we implemented you only look once (YOLO) v3 object detection algorithm available at <https://github.com/pjreddie/darknet> [(1)](https://www.zotero.org/google-docs/?U1Jmn4) to the preprocessed radiographs. Our joint detection model was trained using manually determined bounding boxes, labeled as finger, wrist or foot joints, present in the input images. The pretrained weights from model *darknet53.conv.74* were used as initial weights when training the model. Image annotation tool labelImg (<https://github.com/tzutalin/labelImg>) was used for generating and verifying the bounding boxes prior and during model training.

### ***Joint labeling***

After detecting finger, wrist and foot joints in the input images, we implemented gradient boosting machine (GBM) multi-class classifiers to obtain the correct, more elaborate labels for each joint. R package *gbm* (2.1.8.1) [(2)](https://www.zotero.org/google-docs/?n8WpSQ) was used for training and applying the GBM models. The joint-specific labeling was predicted using features generated based on the coordinates and dimensions of initially detected bounding boxes. These features included:

- area of the bounding box
- area of the bounding box normalized to maximum bounding box area in the current image
- width/height of the bounding box
- center point coordinates of the bounding box
- total number of bounding boxes detected in the current image and proportion of detected bounding boxes in relation to maximum number joints to detect for each joint group
- number of bounding boxes with greater *x* or *y* center point coordinate values compared to the center of the current bounding box
- angle formed between the center point of the current bounding box and outermost detected bounding box (in clockwise direction), measured from a reference point (for finger joints, the reference point was calculated as arithmetic mean of annotation box center points of the wrist joints; for wrist joints, the reference point was calculated as arithmetic mean of annotation box center points of the finger joints; for foot joints, the reference point was the midpoint between the outermost metatarsophalangeal joints)
- absolute distance from the center of the annotation box to the reference point
- relative distance calculated by dividing the current distance with the average distance from the reference point to the outermost finger/toe bounding boxes, depending on the type of current image

In order to train the GBM classifiers using the aforementioned features, all bounding boxes were first labeled manually. Model training was done using 10-fold cross-validation with an interaction depth of 2. The maximum number of trees was set to 500 but for each model the optimal number of iterations leading to the lowest cross-validation error was eventually used. Finally, in the automated scoring pipeline, joint-specific images were generated for score prediction by cropping the joint region from the non-scaled input image according to the labeled bounding boxes.

### ***Score prediction***

The main difference between our original algorithm and AuRA can be found in the score prediction step. In our original algorithm, the SvH scores were predicted simply as discrete classes using joint-specific YOLO v3 models whereas in AuRA, we applied convolutional neural network (CNN) models based on either the DenseNet121 or the DenseNet169 architecture [(3)](https://www.zotero.org/google-docs/?eThd1I) to predict the scores as continuous values.

In order to apply YOLO for score prediction in our original solution, we used an extended implementation of YOLO v3 available in <https://github.com/AlexeyAB/darknet/tree/6056b835eb76b8a078aab18db3e7aba87314f4ce> and modified it further to implement class weights for the different scores during training. We also used *focal_loss* for the YOLO layers in the neural network. Models were trained using a randomly selected 9:1 internal train-test split. To lessen the impact of the bias towards 0, we calculated the score with the second-largest joint numbers in each training dataset and randomly deleted joints with score 0 until the size of both parts was about equal. During prediction, we selected the score with the highest confidence as the estimated score for each joint.

In AuRA, the CNN architecture (DenseNet121 or DenseNet169) with the best performance for each task in the model development cohort was selected for the final algorithm. The prediction of scores as continuous values was achieved by setting a linear activation function for the output layer of the model architecture. The CNN models were implemented in Keras with TensorFlow backend and, to compensate for the limited number of images for training, we applied data augmentation techniques (random zoom within range ±0.1, random rotation within range ±10°) to increase the diversity of the training set. One third of training images were reserved for internal validation during model training. We used the python packages tensorflow (1.15.0), keras (2.3.1), pandas (1.3.5) and numpy (1.18.1) to implement the CNN models.

### ***Score postprocessing***

In the original algorithm, the score postprocessing consisted of imputing the missing scores for undetected joints using median narrowing or erosion scores for each patient and collecting the predictions for all input images into a single csv file. In AuRA, we added additional processing including truncating predicted scores to zero or to the maximum joint- and task-specific score and performing the score imputation for undetected joints within groups of joints based on high Pearson correlation or similar anatomical location (Supplementary Fig. S1A). For remaining joints with missing scores, we assigned the largest possible score under the assumption that the joints missed by YOLO joint detection had typically severe joint damage. Finally, due to systematic underprediction of total SvH scores when calculating the total score as summation of joint-specific scores, we multiplied the obtained raw total scores with a scaling factor of 1.47, determined using the model development cohort, to obtain the final total SvH scores reported by the algorithm (Supplementary Fig. S1B).


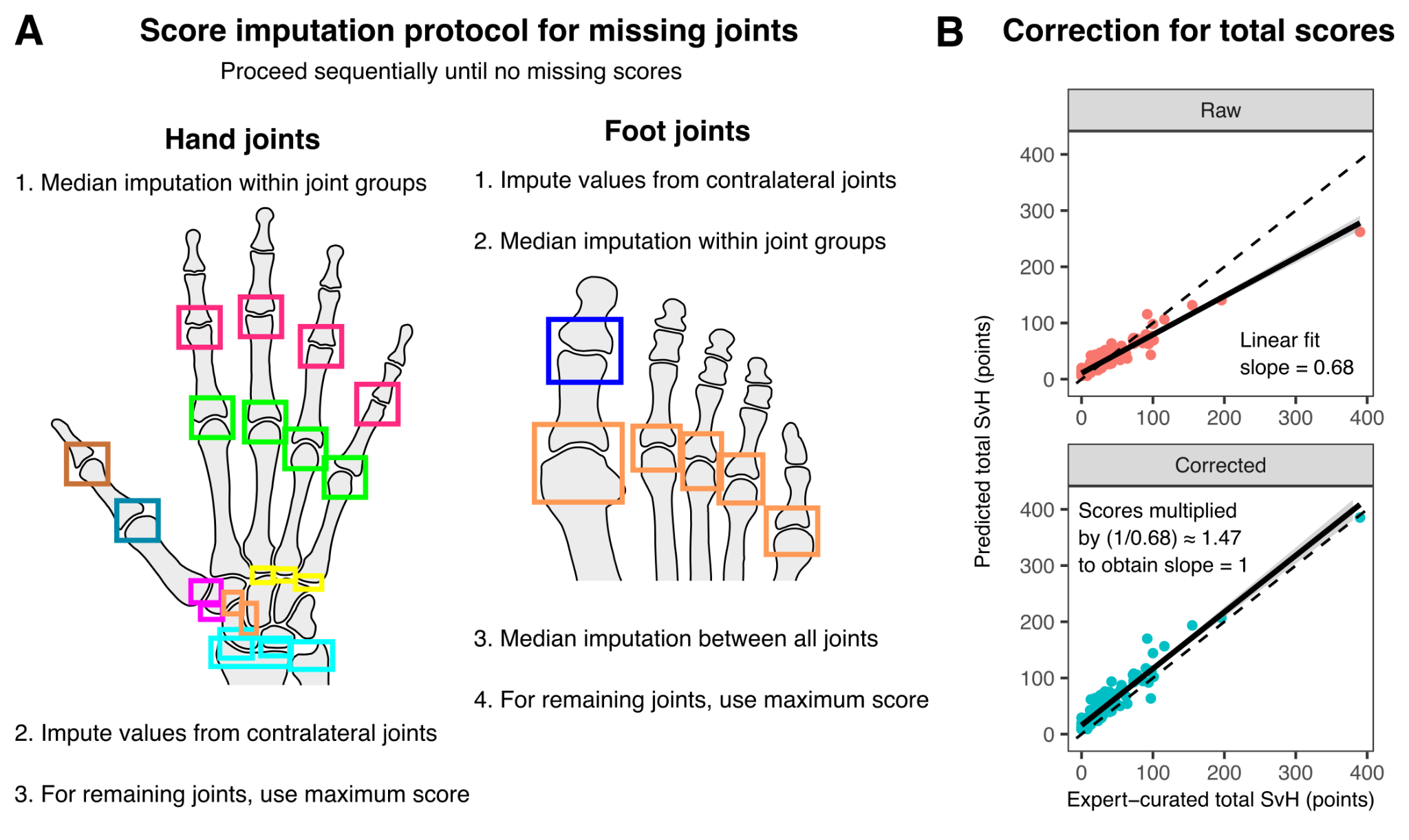


**Supplementary Figure S1.** Postprocessing applied to raw score predictions in AuRA score prediction algorithm. **A)** Imputation scheme to obtain score estimates for joints missed during the joint detection step. **B)** Determination of the correction factor applied to total Sharp van der Heijde (SvH) scores calculated as simple summation over individual joint scores.

### ***Score report***

To facilitate the use of AuRA in clinical practice, we implemented an additional feature to our solution to generate patient-specific reports of the predicted SvH scores. At the beginning of the report, the predicted total SvH score is listed and the preprocessed hand/foot images are shown together with the joints detected for making the predictions. Following the initial summary, all undetected joints are listed to inform the user about the joints for which imputation had to be performed to determine the total score. Finally, all joint-specific scores are listed in descending order along with the corresponding joint-specific images. To simplify the reports, only damaged joints (predicted scores larger than 0.5) were included in the joint-specific listings. The python package matplotlib (3.5.3) was used for generating the reports.

## Joint detection and identification performance

In addition to total SvH scores, we assessed the performance of AuRA in finding the relevant anatomical regions of interest (ROIs) used for SvH scoring in the Turku University Hospital external validation cohort. For this purpose, we compared the ROIs detected and identified using the YOLO v3 and GBM models against manually selected ROIs to calculate the sensitivity of each model as well as the overall algorithm when both models are applied in sequence. To evaluate the effect of joint damage on YOLO v3 performance or the fraction of undetected joints on GBM joint identification performance, the sensitivities were also analyzed within groups with various degrees of bone erosion and joint narrowing as well as with varying joint detection rates, respectively.

# Results

## Study sample

The individual joint-specific SvH scores in the model development cohort, consisting of participants from CLEAR [(4)](https://www.zotero.org/google-docs/?ucRPKE) and TETRAD [(5,6)](https://www.zotero.org/google-docs/?aAT3mo) trials (n=367), and external validation cohort, consisting of patients (n=205) visiting the rheumatology clinic of Turku University Hospital, Finland, can be seen in Supplementary Figs. S2A–B. Nearly all of the joint-specific scores in both cohorts were highly skewed toward zero, resulting in strongly positively skewed distributions of total SvH scores (Supplementary Fig. S2C).


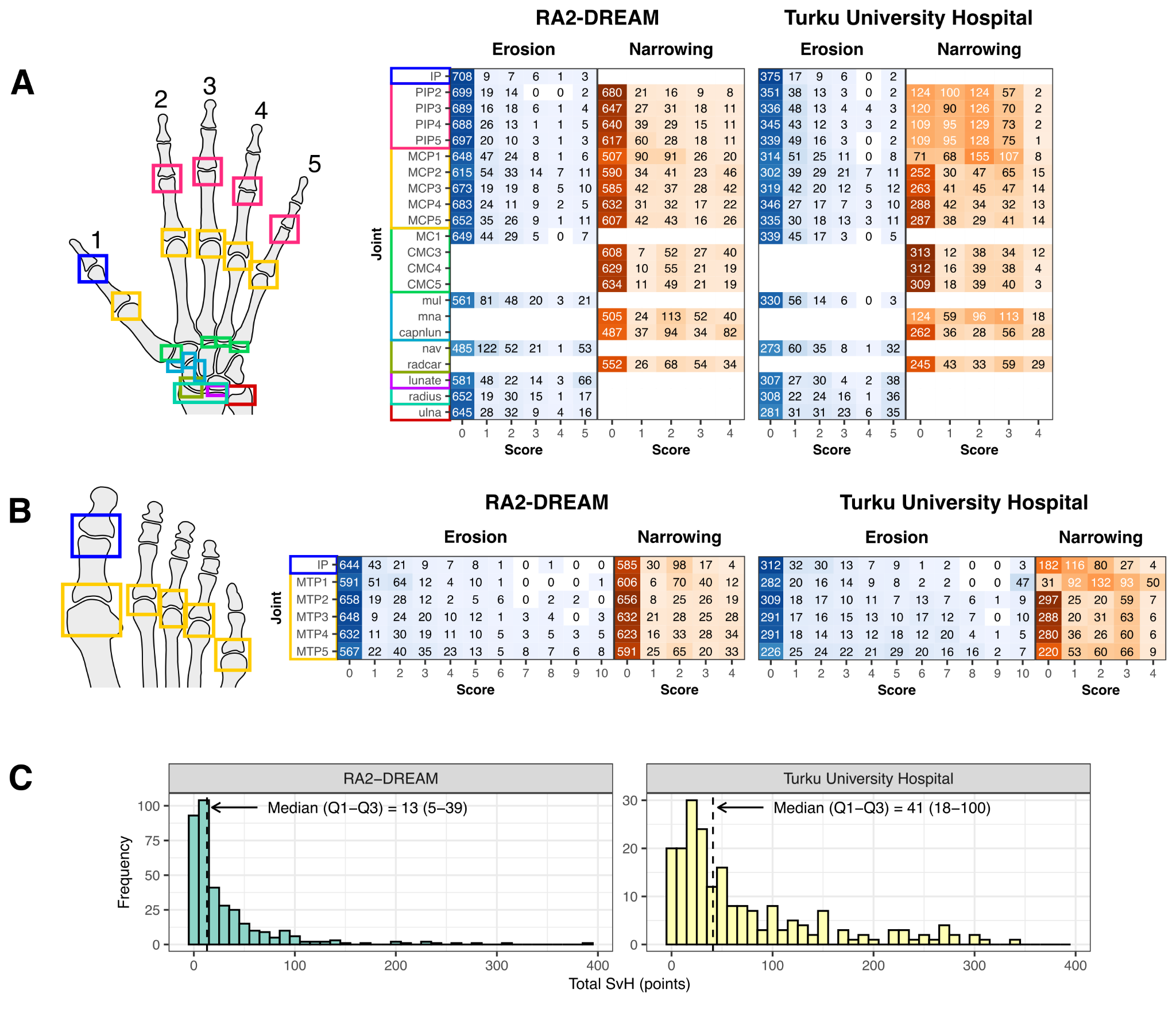


**Supplementary Figure S2.** Distribution of Sharp van der Heijde (SvH) bone erosion and joint narrowing scores in **A)** hand and **B)** foot radiographs as well as **C)** total SvH scores resulting from summation of individual joint scores in the RA2-DREAM challenge model development and Turku University Hospital external validation datasets.

## Joint detection and identification performance

Overall, AuRA extracted the correct anatomical ROIs with a sensitivity of 93.3% (95% confidence interval [CI]: 92.8—93.7) (Supplementary Table S1). In the modeling pipeline, the YOLO v3 joint detection model alone performed with an overall sensitivity of 95.8% (95% CI: 95.4—96.1) which, however, was lower for joints with substantial bone erosion or joint narrowing (Supplementary Fig. S3). The GBM joint identification model, on the other hand, had an overall sensitivity of 98.3% (95% CI: 98.1—98.6). The performance of the GBM model was dependent on YOLO v3 results, *i.e.,* the sensitivity was lower for images with substantial proportion of undetected joints (Supplementary Fig. S4).

**Supplementary Table S1.** Joint detection and labeling performance of AuRA in the Turku University Hospital external validation cohort. Combined performance represents the sensitivity of the algorithm to detect and identify the anatomical regions of interest relevant for determining Sharp van der Heijde scores from raw radiographs.

| Joint | | Joints total | Joint detection performance (YOLOv3) | Joint labeling performance (GBM) | Combined performance (YOLOv3+GBM) |
| --- | --- | --- | --- | --- | --- |
|  |  |  | Sensitivity [%] (95% CI) | Sensitivity [%] (95% CI) | Sensitivity [%] (95% CI) |
| All combined | | 10832 | 95.8 (95.4—96.1) | 98.3 (98.1—98.6) | 93.3 (92.8—93.7) |
|  |  |  |  |  |  |
| Foot joints | | 2447 | 97.5 (96.9—98.1) | 99.0 (98.5—99.3) | 96.5 (95.7—97.2) |
|  | MTP IP | 408 | 96.1 (93.7—97.7) | 98.0 (96.1—99.1) | 95.8 (93.4—97.6) |
|  | MTP1 | 408 | 93.1 (90.2—95.4) | 98.7 (96.9—99.6) | 91.4 (88.3—94.0) |
|  | MTP2 | 408 | 98.8 (97.2—99.6) | 99.2 (97.8—99.8) | 97.3 (95.2—98.6) |
|  | MTP3 | 408 | 98.3 (96.5—99.3) | 99.0 (97.5—99.7) | 97.3 (95.2—98.6) |
|  | MTP4 | 408 | 99.3 (97.9—99.8) | 99.3 (97.9—99.8) | 98.5 (96.8—99.5) |
|  | MTP5 | 407 | 99.8 (98.6—100.0) | 99.5 (98.2—99.9) | 98.8 (97.2—99.6) |
|  |  |  |  |  |  |
| Finger joints | | 4075 | 99.0 (98.7—99.3) | 97.7 (97.2—98.1) | 96.7 (96.1—97.3) |
|  | MCP IP | 408 | 99.0 (97.5—99.7) | 99.8 (98.6—100.0) | 97.8 (95.9—99.0) |
|  | PIP2 | 406 | 99.8 (98.6—100.0) | 98.3 (96.5—99.3) | 98.0 (96.2—99.1) |
|  | PIP3 | 407 | 99.8 (98.6—100.0) | 97.0 (94.9—98.5) | 96.8 (94.6—98.3) |
|  | PIP4 | 407 | 99.5 (98.2—99.9) | 96.8 (94.6—98.3) | 96.8 (94.6—98.3) |
|  | PIP5 | 408 | 98.5 (96.8—99.5) | 97.5 (95.5—98.8) | 95.6 (93.1—97.4) |
|  | MCP1 | 408 | 98.8 (97.2—99.6) | 99.5 (98.2—99.9) | 98.0 (96.2—99.1) |
|  | MCP2 | 407 | 98.3 (96.5—99.3) | 96.5 (94.2—98.1) | 95.6 (93.1—97.4) |
|  | MCP3 | 408 | 98.3 (96.5—99.3) | 95.8 (93.3—97.5) | 94.9 (92.2—96.8) |
|  | MCP4 | 408 | 99.3 (97.9—99.8) | 97.8 (95.8—99.0) | 96.6 (94.3—98.1) |
|  | MCP5 | 408 | 99.0 (97.5—99.7) | 98.0 (96.1—99.1) | 97.3 (95.2—98.6) |
|  |  |  |  |  |  |
| Wrist joints | | 4310 | 91.6 (90.8—92.5) | 98.6 (98.2—99.0) | 88.1 (87.1—89.1) |
|  | CMC3 | 399 | 95.2 (92.7—97.1) | 98.4 (96.6—99.4) | 93.0 (90.0—95.3) |
|  | CMC4 | 405 | 92.8 (89.9—95.2) | 97.1 (94.8—98.5) | 90.4 (87.1—93.1) |
|  | CMC5 | 407 | 91.9 (88.8—94.4) | 97.6 (95.5—98.9) | 89.9 (86.6—92.7) |
|  | mna | 393 | 86.5 (82.7—89.7) | 98.2 (96.1—99.3) | 83.2 (79.1—86.8) |
|  | capnlun | 377 | 77.7 (73.2—81.8) | 99.0 (97.0—99.8) | 75.6 (70.9—79.8) |
|  | radcar/nav | 383 | 95.0 (92.4—97.0) | 97.9 (95.7—99.1) | 83.8 (79.7—87.4) |
|  | ulna | 377 | 96.3 (93.8—98.0) | 99.4 (98.0—99.9) | 95.5 (92.9—97.4) |
|  | radius | 387 | 96.9 (94.6—98.4) | 99.4 (97.9—99.9) | 86.8 (83.0—90.0) |
|  | lunate | 376 | 90.4 (87.0—93.2) | 99.1 (97.4—99.8) | 87.8 (84.0—90.9) |
|  | mul | 401 | 86.3 (82.5—89.5) | 98.8 (97.1—99.7) | 85.5 (81.7—88.8) |
|  | MC1 | 405 | 98.5 (96.8—99.5) | 100.0 (99.1—100.0) | 97.3 (95.2—98.6) |


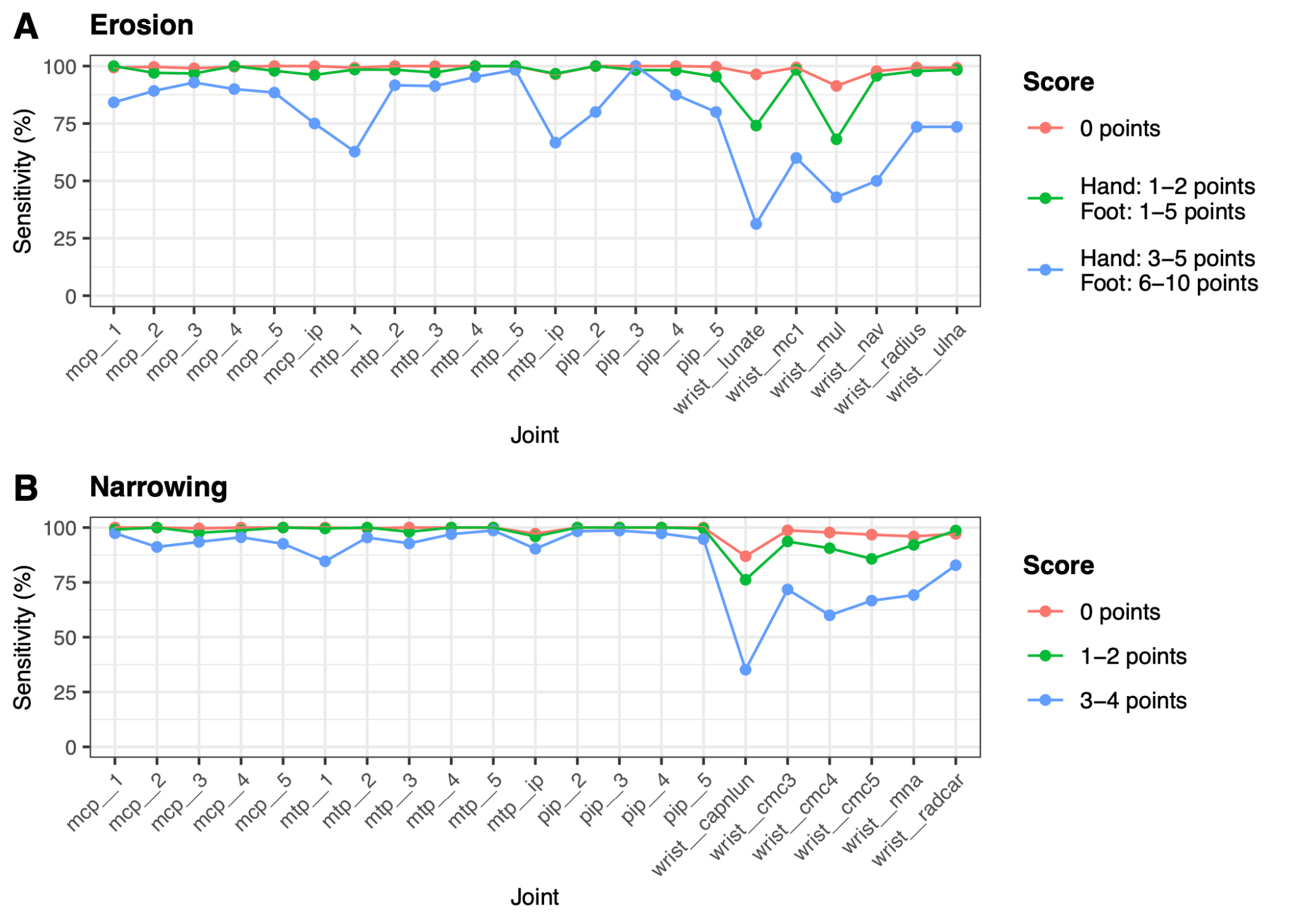


**Supplementary Figure S3.** YOLOv3 joint detection sensitivity in AuRA at different **A)** bone erosion and **B)** joint narrowing score levels in the Turku University Hospital external validation cohort.


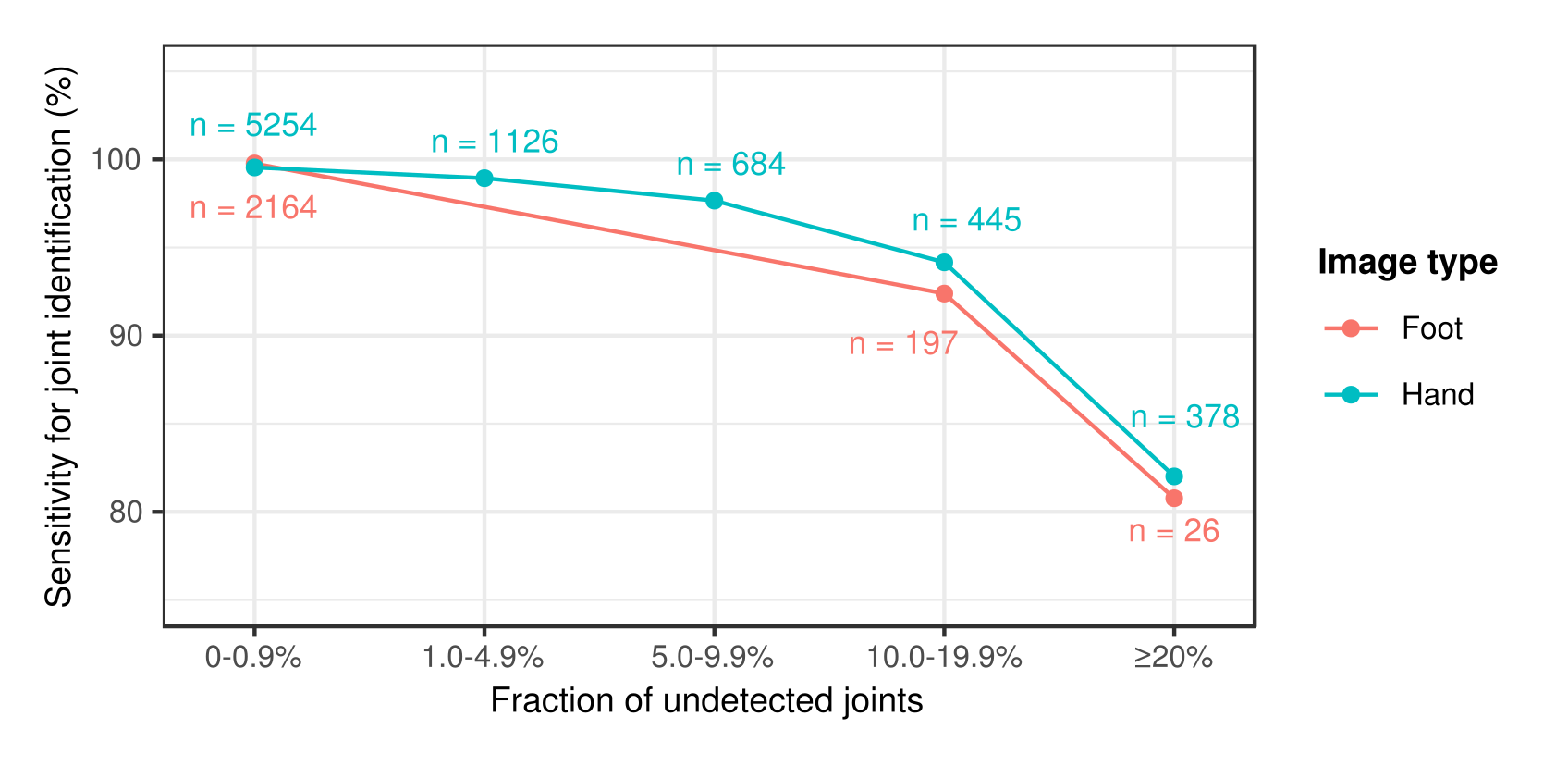


**Supplementary Figure S4.** Joint labeling performance of the GBM model in AuRA versus proportion of undetected joints in images included in the Turku University Hospital external validation cohort.

## Performance of the original algorithm

In predicting the total SvH scores in the external validation cohort, our original automatic scoring algorithm submitted to the RA2-DREAM challenge performed poorly, achieving an RMSE of 80.4 and *R*^2^ of -0.06 (Supplementary Fig. S5). The underperformance occurred most likely due to predicting the joint-specific scores as discrete classes with YOLO v3 instead of continuous values with CNN models as in our new AuRA approach.

##
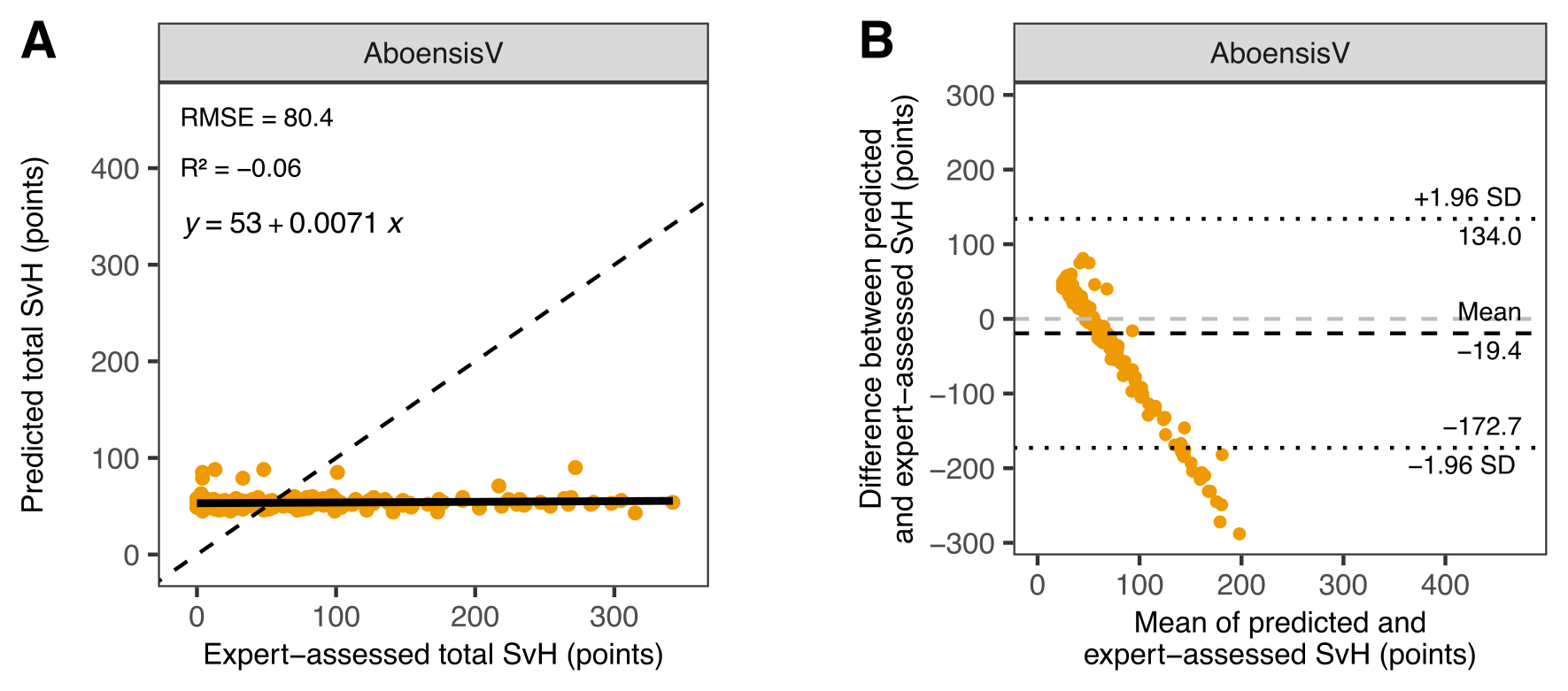


## Supplementary Figure S5. Performance of the original RA2-DREAM challenge algorithm in the Turku University Hospital external validation cohort. The agreement between predicted and expert-assessed Sharp van der Heijde (SvH) scores is demonstrated using A) a scatter plot with linear trend line and B) Bland-Altman plot. RMSE and SD denote root mean square error and standard deviation, respectively.

# References

[1. Redmon J, Divvala S, Girshick R, Farhadi A. You Only Look Once: Unified, Real-Time Object Detection. In: 2016 IEEE Conference on Computer Vision and Pattern Recognition (CVPR) [Internet]. Las Vegas, NV, USA: IEEE; 2016 [cited 2022 Nov 9]. p. 779–88. Available from: http://ieeexplore.ieee.org/document/7780460/](https://www.zotero.org/google-docs/?YvxE5k)

[2. Greenwell B, Boehmke B, Cunningham J, GBM developers. gbm: Generalized Boosted Regression Models. R package version 2.1.8.1 [Internet]. 2022. Available from: https://CRAN.R-project.org/package=gbm](https://www.zotero.org/google-docs/?YvxE5k)

[3. Huang G, Liu Z, van der Maaten L, Weinberger KQ. Densely Connected Convolutional Networks [Internet]. arXiv; 2018 [cited 2022 Nov 9]. Available from: http://arxiv.org/abs/1608.06993](https://www.zotero.org/google-docs/?YvxE5k)

[4. Bridges SL, Causey ZL, Burgos PI, Huynh BQN, Hughes LB, Danila MI, et al. Radiographic severity of rheumatoid arthritis in African Americans: results from a multicenter observational study. Arthritis Care Res (Hoboken). 2010 May;62(5):624–31.](https://www.zotero.org/google-docs/?YvxE5k)

[5. Ormseth MJ, Yancey PG, Solus JF, Bridges SL, Curtis JR, Linton MF, et al. Effect of drug therapy on net cholesterol efflux capacity of HDL-enriched serum in rheumatoid arthritis. Arthritis Rheumatol. 2016 Sep;68(9):2099–105.](https://www.zotero.org/google-docs/?YvxE5k)

[6. Ptacek J, Hawtin RE, Sun D, Louie B, Evensen E, Mittleman BB, et al. Diminished cytokine-induced Jak/STAT signaling is associated with rheumatoid arthritis and disease activity. PLoS One. 2021;16(1):e0244187.](https://www.zotero.org/google-docs/?YvxE5k)
